# Supplementary material for: Impact of hydronium ions on the Pd-catalyzed furfural hydrogenation
Source: Nat Commun. 2022 Nov 22;13:7154. doi: 10.1038/s41467-022-34608-8 (PMC9684141; doi:10.1038/s41467-022-34608-8)
Supplement: Supplementary file 1 — Supplementary Information [file 41467_2022_34608_MOESM1_ESM.pdf]

Impact of hydronium ions on the Pd-catalyzed furfural hydrogenation

Iris K.M. Yu[1,2], Fuli Deng[1], Xi Chen[1], Guanhua Cheng[1,3], Yue Liu[1,4], Wei Zhang[1,4,*] and Johannes A. Lercher[1,5,*]

[1]Department of Chemistry and Catalysis Research Center, Technische Universität München, Lichtenbergstrasse 4, 85748 Garching, Germany.

[2]Research Institute for Future Food and Department of Applied Biology and Chemical Technology, The Hong Kong Polytechnic University, Hung Hom, Kowloon, Hong Kong, China.

[3]Key Laboratory for Liquid-Solid Structural Evolution and Processing of Materials (Ministry of Education), School of Materials Science and Engineering, Shandong University, Jingshi Road 17923, Jinan 250061, P.R. China.

[4]Shanghai Key Laboratory of Green Chemistry and Chemical Processes, School of Chemistry and Molecular Engineering, East China Normal University, Shanghai, 200062, China.

[5]Institute for Integrated Catalysis, Pacific Northwest National Laboratory, P.O. Box 999, Richland, WA 99352, United States.

*Corresponding authors: Wei Zhang (weizhang@chem.ecnu.edu.cn); Johannes A. Lercher (johannes.lercher@tum.de)

Supplementary Information

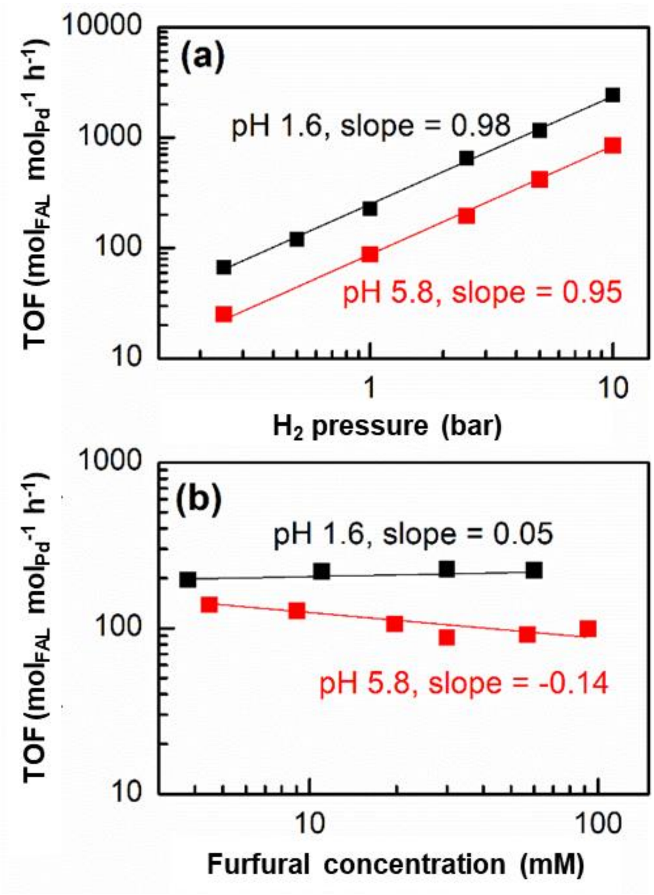

Figure S1. Reaction orders of furfural hydrogenation with respect to (a) H_2 pressure (with 30 mM furfural) and (b) furfural concentrations (at 1 bar H_2). The reaction was performed with Pd/C at room temperature in 0.1 M phosphate buffer solution. Source data are provided as a Source Data file.

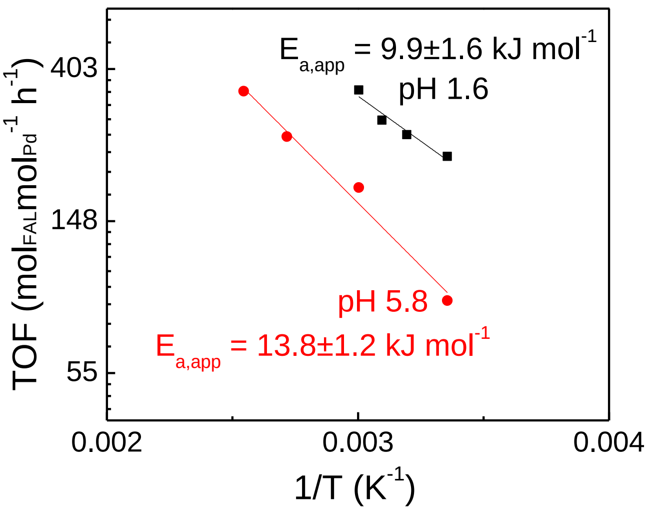

Figure S2. Arrhenius plot of furfural hydrogenation for the calculation of apparent activation energy ($E_{a,app}$). The reaction was performed with 30 mM furfural in 0.1 M phosphate buffer solution with 10 mg Pd/C at 1 bar H_2. Source data are provided as a Source Data file.

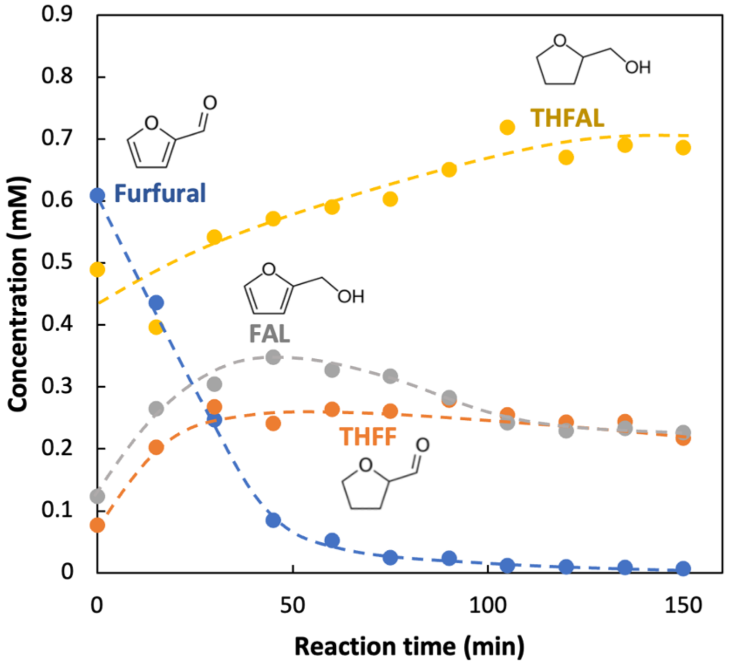

Figure S3. Furfural and product concentrations as a function of reaction time during hydrogenation at a low initial furfural concentration of 1.3 mM. The reaction was performed in 0.1 M phosphate buffer solution (pH 4.5) with 1.4 mg Pd/C at 1 bar H_2 and room temperature. Source data are provided as a Source Data file.

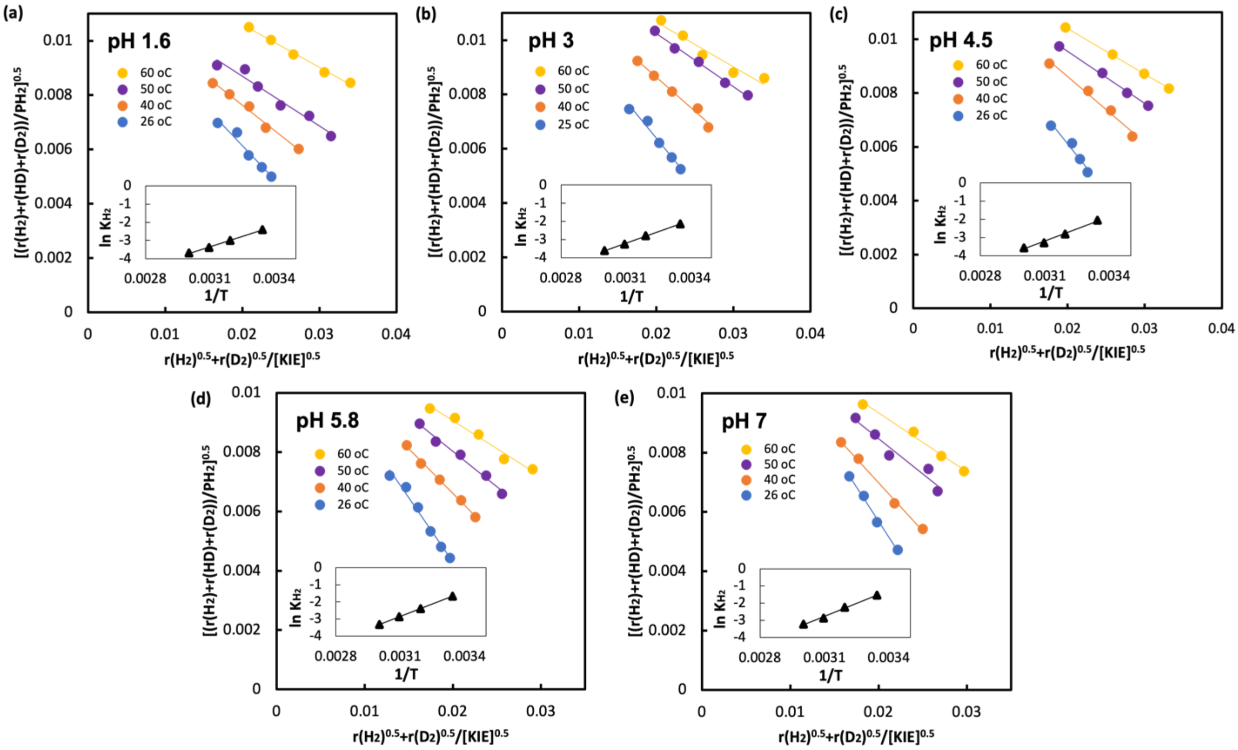

Figure S4. H/D exchange kinetics over 100 mg Pd/C at different pressures and temperatures, at (a) pH 1.6, (b) pH 3, (c) pH 4.5, (d) pH 5.8 and (e) pH 7, plotted according to **Table S3**. The insets show the natural logarithm of equilibrium constant (K_{H_2}) as a function of the inverse of temperature (T) for the calculation of enthalpy change using the Van 't Hoff equation. Source data are provided as a Source Data file.

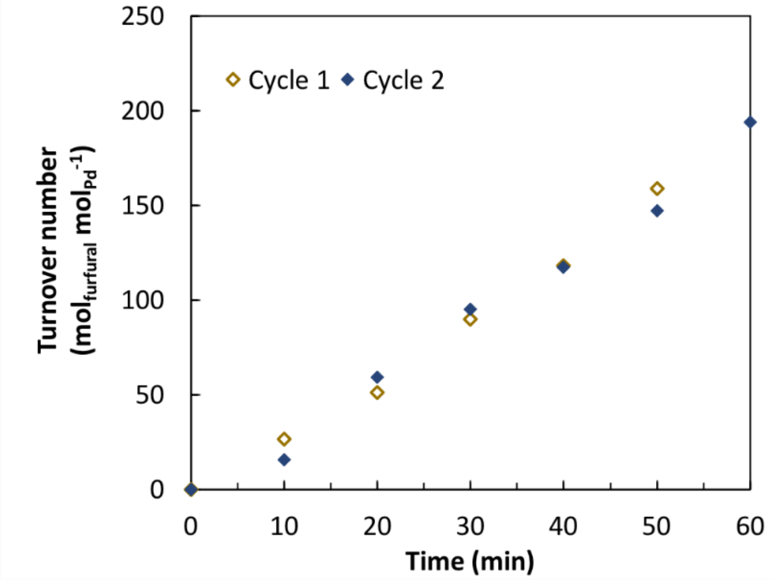

Figure S5. Turnover number of furfural hydrogenation in the recycling test. The reaction was performed with 30 mM furfural in 0.1 M phosphate buffer solution (pH 1.6). After the first run, the separated catalyst was separated by rinsed with water, oven-dried and directly used in the second cycle. Source data are provided as a Source Data file.

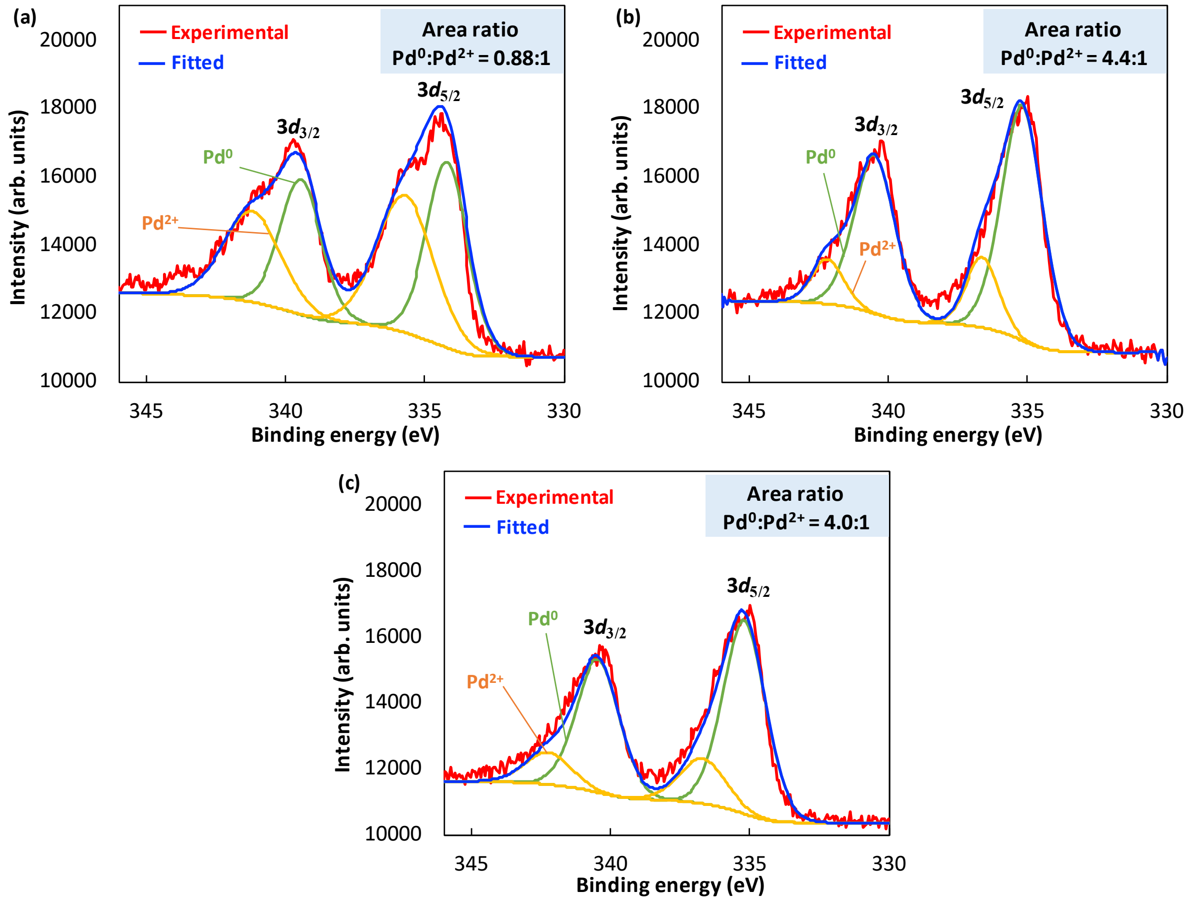

Figure S6. XPS curves and fittings of (a) as-received Pd/C, (b) Pd/C after *in-situ* aqueous-phase reduction at 30 bar H_2 and room temperature for 30 min, and (c) Pd/C recovered from catalytic furfural hydrogenation of 30 mM furfural in 0.1 M phosphate buffer solution (pH 4.5) at 1 bar H_2 and room temperature for 1h. Source data are provided as a Source Data file.

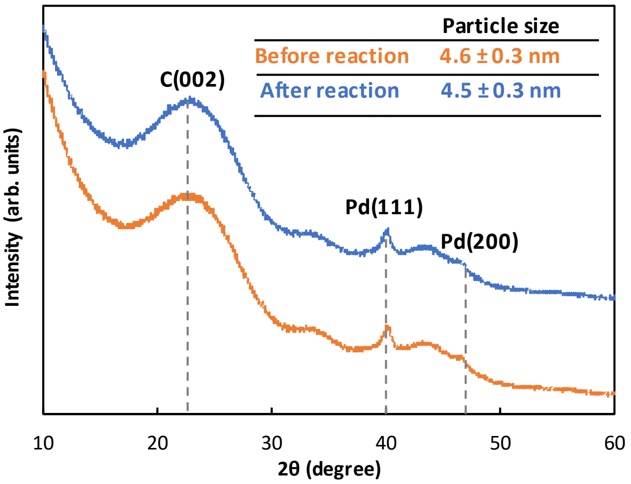

Figure S7. XRD patterns of *in-situ* reduced Pd/C before (orange color) and after (blue color) catalytic furfural hydrogenation. In situ reduction condition: 0.1 M phosphate buffer solution (pH 4.5), 30 bar H_2 and room temperature for 30 min. Furfural hydrogenation condition: 30 mM furfural in 0.1 M phosphate buffer solution (pH 4.5) at 1 bar H_2 and room temperature for 1h. Source data are provided as a Source Data file.

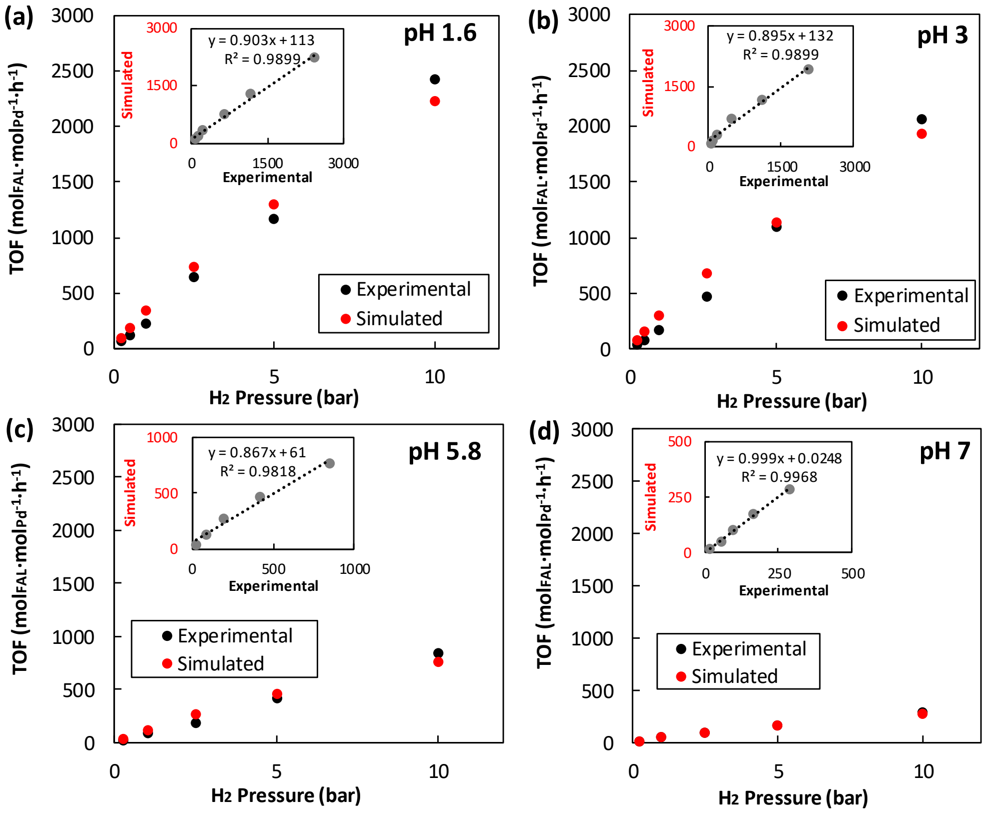

Figure S8. Simulated TOFs of furfural hydrogenation using Eqn. (2) (main text) and experimental TOFs with 30 mM furfural in 0.1 M phosphate buffer solution with 10 mg Pd/C at 0.25-10 bar H_2 and room temperature at pH (a) 1.6, (b) 3, (c) 5.8, and (d) 7. The parity plots are presented as the insets. Source data are provided as a Source Data file.

Table S1. Selectivity of furfuryl alcohol (FAL), tetrahydrofurfural (THFF), and tetrahydrofurfuryl alcohol (THFAL) as well as carbon balance at furfural conversions of around 20-35%.*

pH	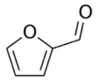 Furfural conversion (mol%)	Product selectivity (mol%)			C balance (mol%)
		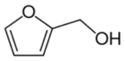 FAL	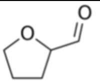 THFF	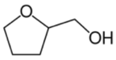 THFAL	
1.6	19.4	88.8	3.6	1.5	98.8
3.0	21.0	92.1	5.1	3.5	100.2
4.5	29.7	94.5	5.3	4.9	101.4
5.8	35.5	83.9	7.4	7.6	99.6
7.0	22.6	81.9	11.9	6.2	100.0

* The reaction was performed with 30 mM furfural in 0.1 M phosphate buffer solution with 10 mg Pd/C at 1 bar H_2 and room temperature.
Selectivity = Yield / Conversion.
C balance = (Concentrations of products and unconverted Furfural) / Concentration of initial Furfural)

Table S2. Selectivity of furfuryl alcohol (FAL), tetrahydrofurfural (THFF), and tetrahydrofurfuryl alcohol (THFAL) from the hydrogenation of furfural at low and high concentration.*

pH	Initial furfural concentration (mM)	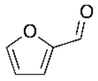 Furfural conversion (mol%)	Product selectivity (mol%)		
			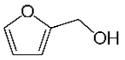 FAL	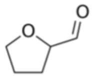 THFF	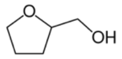 THFAL
4.5	1.3	66.5	30.7	23.5	45.9
		96.0	26.2	21.1	47.3
4.5	30	34.0	74.2	10.7	5.6
5.8	30	50.9	83.1	12.1	7.8

* The reaction was performed in 0.1 M phosphate buffer solution at 1 bar H_2 and room temperature.

Table S3. Summary of the kinetic analysis method developed by Yang et al.[1] for the measurement of H_2 adsorption equilibrium constant (K_{H_2}).

Reaction	H_2 (g) + D_2O (l) $\xrightarrow{Pd}$ HD (g) + HDO (l)<br>H_2 (g) + 2D_2O (l) $\xrightarrow{Pd}$ D_2 (g) + 2HDO (l)
Equation	$(r_{H_2} + r_{HD} + r_{D_2})^{0.5} \cdot P_{H_2}^{-0.5} = k_{H_2}^{0.5} - (r_{H_2}^{0.5} + r_{D_2}^{0.5} \cdot [KIE]_{D_2}^{-0.5}) \cdot K_{H_2}^{0.5}$
Data acquisition	P_{H_2} is the applied H_2 pressure;<br>r_{HD} and r_{D_2} are measured as HD and D_2 formation rate, respectively;<br>KIE refers to the kinetic isotope effect;<br>Calculation of H_2 formation rate (r_{H_2}) = $\frac{[KIE]_{D_2} \cdot r_{HD}^2}{4[KIE]_{HD}^2 \cdot r_{D_2}}$
Plot	$(r_{H_2} + r_{HD} + r_{D_2})^{0.5} \cdot P_{H_2}^{-0.5}$ vs. $(r_{H_2}^{0.5} + r_{D_2}^{0.5} \cdot [KIE]_{D_2}^{-0.5})$<br>gives a linear curve: K_{H_2} = slope^2

Note S1. Thermodynamic cycle for furfural adsorption

A thermodynamic cycle is constructed considering furfural solvation and adsorption in gas and aqueous phases (**Figure 4** in main text). The details of obtaining $\Delta H^0_{F\ solv}$ and $\Delta H^0_{H_2O\ ads,l}$ are given in **Table S4** and **S5**, respectively. For simplicity, the difference between bond energy (ΔU) and enthalpy (ΔH) in gas-forming steps is considered negligible (ΔU = ΔH + RT, and RT = 2.5 kJ mol^{-1} at 298 K is omitted).

Table S4. Enthalpy change of the solvation of furfural(g).

Step	Enthalpy	Quantification
Furfural solvation:<br>furfural(g)→furfural(aq)	$\Delta H^0_{F\ solv}$	Calculated using the van't Hoff equation:<br>$$\frac{d\ lnk_H}{d\ (1/T)} = -\frac{\Delta H^0_{F\ solv}}{R}$$<br>where $\frac{d\ lnk_H}{d\ (1/T)}$ = 6100 (from refs[2,3]) as the Henry's law constant temperature dependence, R is the gas constant

Note that $\Delta H^0_{H_2O\ ads,l}$ for the adsorption of H_2O molecules on Pd is normalized by the furfural footprint, *i.e.*, the Pd area occupied by one adsorbed furfural. We consider the displacement of 6.5 H_2O molecules per adsorbed furfural molecule, similar to adsorption of phenol that also has seven heavy atoms (O and C) in total.[4] In essence, the adsorption of water breaks down into sequential bond formation and dissociation steps to estimate $\Delta H^0_{H_2O\ ads,l}$ (**Figure S9**).

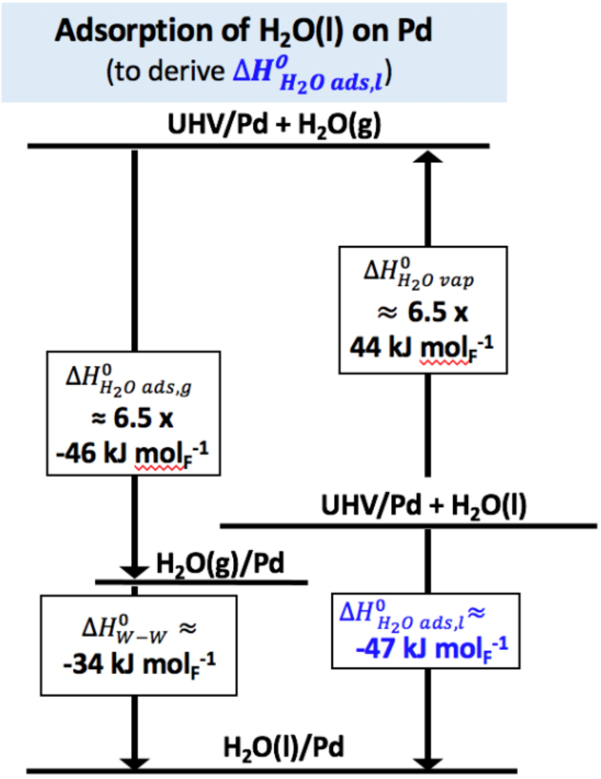

Figure S9. Thermodynamic cycle of adsorption of water on Pd.

Table S5. Enthalpy changes for the steps in water adsorption on Pd (normalized by furfural footprint).

Step	Enthalpy	Quantification
Vaporization of H_2O(l)	$\Delta H^{o}_{H_2O\ vap}$	6.5 × water vaporization enthalpy at 298 K from ref [5]
Adsorption of H_2O(g)	$\Delta H^{o}_{H_2O\ ads,g}$	6.5 × water adsorption enthalpy at 298 K from ref [6]
Formation of water−water bond from two liquid/vacuum interfaces	ΔH^{o}_{W-W}	Calculated using water surface energy ($\gamma_{H2O(l)}$ = 0.073 J m^{-2} from refs [4,7]), the area of one adsorbed furfural molecule (A_F) on Pd in aqueous phase, and Avogadro's number (N_A). $$\Delta H^{o}_{W-W} = -2\gamma_{H_2O(l)} \cdot A_F \cdot N_A$$ $$A_F = \frac{\sqrt{3} \cdot (a_{lat})^2}{2 \cdot n \cdot d}$$ where *n* = 2 as the number of Pd atoms in (111), a_{lat} = 3.89 Å as the lattice constant of Pd (ref [8]), and d = 1/6 monolayer (ML) as the adsorbate density (*i.e.*, one furfural adsorbed on six Pd atoms). Then, $A_F = 3.87 \times 10^{-19}\ m^2$
Adsorption of H_2O(l)	$\Delta H^{o}_{H_2O\ ads,l}$	Sum of $\Delta H^{o}_{H_2O\ vap}$, $\Delta H^{o}_{H_2O\ ads,g}$, and ΔH^{o}_{W-W}

Note S2. Rate equation derivation for furfural hydrogenation

Table S6. Elementary steps in furfural hydrogenation include:

(1) H_2 adsorption	$H_2 + 2 * \rightleftharpoons 2 H*$	$K^o_{H_2}$
(2) Furfural adsorption	$F + * \rightleftharpoons F*$	K^o_F
(3) 1st H addition	$F* + H* \rightleftharpoons FH* + *$	K^o_1
	$F* + H^+ + e^- \rightleftharpoons FH*$	$K^o_{1,PCET}$
	$H* \rightleftharpoons H^+ + e^- + *$	$K^o_{1,Volmer}$
(4) 2nd H addition	$FH* + H* \rightarrow FH_2 + 2 *$	k_2

We develop the kinetic model on the basis of 1) rds following the Langmuir Hinshelwood mechanism, 2) non-competitive adsorption (for the kinetic regime of interest in this work), and 3) 2nd H addition to furfural (step 4) being the rds. Furfural mass transport is not rate-limiting in view of the 0th reaction order of furfural (**Figure S1b**). A supplementary experiment was performed by reaction of furfural in the presence of FAL. The result shows that the presence of FAL of equivalent concentration did not significantly impact furfural conversion (**Figure S10**), suggesting that product desorption is not rate-limiting. As the last step, FAL desorption can be excluded from the kinetic model. In addition, we measured the adsorption rate of H_2 on Pd/C through the H/D exchange experiment, i.e., H_2 (g) + D_2O (l) → HD (g) + HDO (l), 0.5 H_2 (g) + D_2O (l) → 0.5 D_2 (g) + HDO (l), (TOF > 12,000 h^{-1}; **Figure 5**). This indicates that the diffusion rate of H_2 and the collision frequency of H_2 on Pd is at least 12,000 h^{-1}. Given such a high rate compared to furfural hydrogenation (TOF < 2,500 h^{-1}; **Figure 1**), it is very unlikely that diffusion accounts for the first order dependence in H_2 and is thus excluded from the kinetic model.

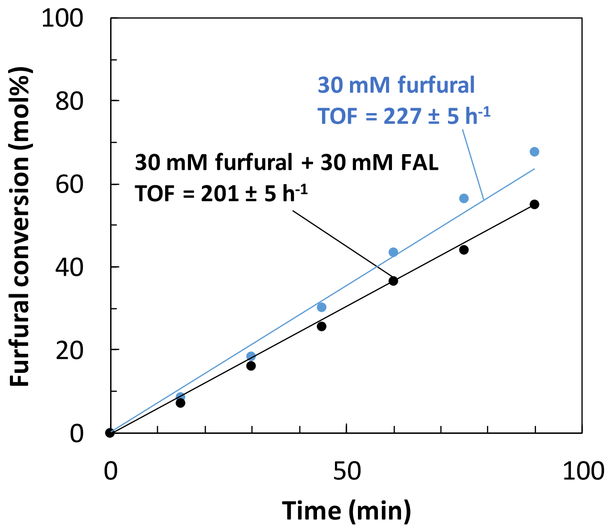

Figure S10. Furfural conversion and turnover frequency (TOF) (mol mol_{Pd}^{-1} h^{-1}) in the absence and presence of FAL (30 mM). The reaction was performed with 30 mM furfural in 0.1 M phosphate buffer solution at pH 1.6 with 10 mg Pd/C at 1 bar H_2 and room temperature.

H_2 adsorption, furfural adsorption, and the 1st H addition are quasi-equilibrated under the experimental conditions, of which the equilibrium constants (K) are shown below. Note that in Step 3 (Table S6), our KIE study presents no evidence of the H addition pathway (Figure 2, main text). Nevertheless, it remains sensible to consider only the surface reaction and its

equilibrium constant K_1^o in our kinetic model because all species in this step are in the state of quasi-equilibrium.

$$K_{H_2}^{0.5} = \frac{\theta_H}{P_{H_2}^{0.5}\theta_{*,1}} \qquad \text{(Eqn. S1a)}$$

$$K_F = \frac{\theta_F}{C_F\theta_{*,2}} \qquad \text{(Eqn. S1b)}$$

$$K_1 = \frac{\theta_{FH}\theta_{*,1}}{\theta_F\theta_H} \qquad \text{(Eqn. S1c)}$$

They are rewritten to derive the expressions for the coverages of dissociatedly adsorbed H (θ_H), molecularly adsorbed furfural (θ_F), and H-added furfural (θ_{FH}), in terms of K, H_2 pressure (P_{H_2}), furfural concentration (C_F), and empty site coverages ($\theta_{*,1}$& $\theta_{*,2}$ for hydrogen and furfural, respectively, for non-competitive adsorption).

$$\theta_H = K_{H_2}^{0.5}P_{H_2}^{0.5}\theta_{*,1} \qquad \text{(Eqn. S2a)}$$

$$\theta_F = K_F C_F\theta_{*,2} \qquad \text{(Eqn. S2b)}$$

Combining Eqn. S1c with Eqn. S2a&b:

$$\theta_{FH} = \frac{K_1\theta_H\theta_F}{\theta_{*,1}} = K_1K_FC_FK_{H_2}^{0.5}P_{H_2}^{0.5}\theta_{*,2} \qquad \text{(Eqn. S2c)}$$

As for non-competitive adsorption, the sum of the coverages of surface species:

$$\theta_H + \theta_{*,1} = 1 \qquad \text{(Eqn. S3a)}$$

$$\theta_F + \theta_{FH} + \theta_{*,2} = 1 \qquad \text{(Eqn. S3b)}$$

Combining Eqn. S2a and S3a gives:

$$K_{H_2}^{0.5}P_{H_2}^{0.5}\theta_{*,1} + \theta_{*,1} = 1$$

$$\theta_{*,1} = \frac{1}{K_{H_2}^{0.5}P_{H_2}^{0.5}+1} \qquad \text{(Eqn. S4a)}$$

Combining Eqn. S2b&c and S3b gives:

$$K_FC_F\theta_{*,2} + K_1K_FC_FK_{H_2}^{0.5}P_{H_2}^{0.5}\theta_{*,2} + \theta_{*,2} = 1$$

$$\theta_{*,2} = \frac{1}{K_FC_F+K_1K_FC_FK_{H_2}^{0.5}P_{H_2}^{0.5}+1} \qquad \text{(Eqn. S4b)}$$

Combining Eqn. S2 and S4, the coverages of the surface species are expressed as follows.

$$\theta_H = K_{H_2}^{0.5}P_{H_2}^{0.5}\theta_{*,1} = \frac{K_{H_2}^{0.5}P_{H_2}^{0.5}}{K_{H_2}^{0.5}P_{H_2}^{0.5}+1} \qquad \text{(Eqn. S5a)}$$

$$\theta_F = K_FC_F\theta_{*,2} = \frac{K_FC_F}{K_FC_F+K_1K_FC_FK_{H_2}^{0.5}P_{H_2}^{0.5}+1} \qquad \text{(Eqn. S5b)}$$

$$\theta_{FH} = K_1 K_F C_F K_{H_2}^{0.5} P_{H_2}^{0.5} \theta_{*,2} = \frac{K_1 K_F C_F K_{H_2}^{0.5} P_{H_2}^{0.5}}{K_F C_F + K_1 K_F C_F K_{H_2}^{0.5} P_{H_2}^{0.5} + 1} \quad \text{(Eqn. S5c)}$$

The rate equation for the rds (*i.e.*, the 2nd H addition) is derived by substituting the coverages with Eqn S5a&c.

$$r = k_2 \theta_H \theta_{FH}$$

$$= k_2 \cdot \frac{K_{H_2}^{0.5} P_{H_2}^{0.5}}{K_{H_2}^{0.5} P_{H_2}^{0.5} + 1} \cdot \frac{K_1 K_F C_F K_{H_2}^{0.5} P_{H_2}^{0.5}}{K_F C_F + K_1 K_F C_F K_{H_2}^{0.5} P_{H_2}^{0.5} + 1}$$

$$= \frac{k_2 K_{H_2} P_{H_2} K_1 K_F C_F}{(K_{H_2}^{0.5} P_{H_2}^{0.5} + 1)(K_F C_F + K_1 K_F C_F K_{H_2}^{0.5} P_{H_2}^{0.5} + 1)} \quad \text{(Eqn. S6a)}$$

As K_F >> 1 >> K_{H_2} according to our measurements (**Table 3**, main text), denominator in Eqn. S6a $[(K_{H_2}^{0.5} P_{H_2}^{0.5} + 1)(K_F C_F + K_1 K_F C_F K_{H_2}^{0.5} P_{H_2}^{0.5} + 1)]$ can be simplified to $K_F C_F$. Then,

$$r \approx \frac{k_2 K_{H_2} P_{H_2} K_1 K_F C_F}{K_F C_F} \approx k_2 K_{H_2} P_{H_2} K_1 \quad \text{(Eqn. S6b)}$$

Note S3. Derivation of equations for reaction orders

1. Reaction order in H_2

H_2 reaction order = $\frac{\partial \ln r}{\partial \ln P_{H_2}}$

$$= \frac{\partial P_{H_2}}{\partial \ln P_{H_2}} \cdot \frac{\partial \ln r}{\partial P_{H_2}}$$

$$= P_{H_2} \cdot \frac{\partial \ln r}{\partial P_{H_2}}$$

$$= P_{H_2} \cdot \frac{\partial}{\partial P_{H_2}} \ln \left[\frac{k_2 K_{H_2} P_{H_2} K_1 K_F C_F}{(K_{H_2}^{0.5} P_{H_2}^{0.5} + 1)(K_F C_F + K_1 K_F C_F K_{H_2}^{0.5} P_{H_2}^{0.5} + 1)} \right]$$

$$= P_{H_2} \cdot \frac{\partial}{\partial P_{H_2}} \left[\ln\left(k_2 K_{H_2} P_{H_2} K_1 K_F C_F\right) - \ln\left(K_{H_2}^{0.5} P_{H_2}^{0.5} + 1\right) - \ln\left(K_F C_F + K_1 K_F C_F K_{H_2}^{0.5} P_{H_2}^{0.5} + 1\right) \right]$$

$$= P_{H_2} \cdot \left(\frac{1}{P_{H_2}} - \frac{0.5 K_{H_2}^{0.5} P_{H_2}^{-0.5}}{K_{H_2}^{0.5} P_{H_2}^{0.5} + 1} - \frac{0.5 K_1 K_F C_F K_{H_2}^{0.5} P_{H_2}^{-0.5}}{K_F C_F + K_1 K_F C_F K_{H_2}^{0.5} P_{H_2}^{0.5} + 1} \right)$$

$$= 1 - \frac{0.5 K_{H_2}^{0.5} P_{H_2}^{0.5}}{K_{H_2}^{0.5} P_{H_2}^{0.5} + 1} - \frac{0.5 K_1 K_F C_F K_{H_2}^{0.5} P_{H_2}^{0.5}}{K_F C_F + K_1 K_F C_F K_{H_2}^{0.5} P_{H_2}^{0.5} + 1} \quad \text{(Eqn. S7)}$$

By substituting Eqn. S5a&c into Eqn. S7, the reaction order in H_2:

$$n_{H_2} = 1 - 0.5(\theta_H + \theta_{FH}) \quad \text{(Eqn. S8)}$$

2. Reaction order in furfural

Furfural reaction order = $\frac{\partial \ln r}{\partial \ln C_F}$

$$= \frac{\partial C_F}{\partial \ln C_F} \cdot \frac{\partial \ln r}{\partial C_F}$$

$$= C_F \cdot \frac{\partial \ln r}{\partial C_F}$$

$$= C_F \cdot \frac{\partial}{\partial C_F} \ln \left[\frac{k_2 K_{H_2} P_{H_2} K_1 K_F C_F}{(K_{H_2}^{0.5} P_{H_2}^{0.5} + 1)(K_F C_F + K_1 K_F C_F K_{H_2}^{0.5} P_{H_2}^{0.5} + 1)} \right]$$

$$= C_F \cdot \frac{\partial}{\partial C_F} \left[\ln\left(k_2 K_{H_2} P_{H_2} K_1 K_F C_F\right) - \ln\left(K_{H_2}^{0.5} P_{H_2}^{0.5} + 1\right) - \ln\left(K_F C_F + K_1 K_F C_F K_{H_2}^{0.5} P_{H_2}^{0.5} + 1\right) \right]$$

$$= C_F \cdot \left(\frac{1}{C_F} - \frac{K_F + K_1 K_F K_{H_2}^{0.5} P_{H_2}^{0.5}}{K_F C_F + K_1 K_F C_F K_{H_2}^{0.5} P_{H_2}^{0.5} + 1} \right)$$

$$= C_F \cdot \left(\frac{1}{C_F} - \frac{K_F + K_1 K_F K_{H_2}^{0.5} P_{H_2}^{0.5}}{K_{H_2}^{0.5} P_{H_2}^{0.5} + K_F C_F + K_1 K_F C_F K_{H_2}^{0.5} P_{H_2}^{0.5} + 1} \right)$$

$$= 1 - \frac{K_F C_F + K_1 K_F C_F K_{H_2}^{0.5} P_{H_2}^{0.5}}{K_{H_2}^{0.5} P_{H_2}^{0.5} + K_F C_F + K_1 K_F C_F K_{H_2}^{0.5} P_{H_2}^{0.5} + 1} \quad \text{(Eqn. S9)}$$

By substituting Eqn. S5b&c into Eqn. S9, the reaction order in furfural:

$$n_F = 1 - (\theta_F + \theta_{FH}) \quad \text{(Eqn. S10)}$$

Note S4. Equations for kinetic model with the first H addition as the rds

If the 1st H addition (Step 3 in **Table S6**) is the rds with the rate constant k_1, the rate equation and coverage expressions:

$$r = k_1\theta_H\theta_F \qquad \text{(Eqn. S11)}$$

$$\theta_H + \theta_{*,1} = 1 \quad \text{and} \quad \theta_F + \theta_{*,2} = 1 \qquad \text{(Eqn. S12)}$$

Substituting Eqn. S2a&b into Eqn. S12

$$\theta_{*,1} = \frac{1}{K_{H_2}^{0.5}P_{H_2}^{0.5}+1} \quad \text{and} \quad \theta_{*,2} = \frac{1}{K_F C_F+1} \qquad \text{(Eqn. S13)}$$

Combining Eqn. S2a&b and S13, the coverage expressions become:

$$\theta_H = \frac{K_{H_2}^{0.5}P_{H_2}^{0.5}}{K_{H_2}^{0.5}P_{H_2}^{0.5}+1} \quad \text{and} \quad \theta_F = \frac{K_F C_F}{K_F C_F+1} \qquad \text{(Eqn. S14)}$$

Then the rate equation (Eqn. S11) becomes:

$$r = \frac{k_1 K_{H_2}^{0.5}P_{H_2}^{0.5}K_F C_F}{(K_{H_2}^{0.5}P_{H_2}^{0.5}+1)(K_F C_F+1)} \qquad \text{(Eqn. S15)}$$

Reaction order in H2 = $\frac{\partial \ln r}{\partial \ln P_{H_2}}$

$$= \frac{\partial P_{H_2}}{\partial \ln P_{H_2}} \cdot \frac{\partial \ln r}{\partial P_{H_2}} = P_{H_2} \cdot \frac{\partial \ln r}{\partial P_{H_2}} = P_{H_2} \cdot \frac{\partial}{\partial P_{H_2}} \ln\left[\frac{k_1 K_{H_2}^{0.5}P_{H_2}^{0.5}K_F C_F}{(K_{H_2}^{0.5}P_{H_2}^{0.5}+1)(K_F C_F+1)}\right]$$

$$= P_{H_2} \cdot \frac{\partial}{\partial P_{H_2}}\left[\ln\left(k_1 K_{H_2}^{0.5}P_{H_2}^{0.5}K_F C_F\right) - \ln\left(K_{H_2}^{0.5}P_{H_2}^{0.5}+1\right) - \ln(K_F C_F+1)\right]$$

$$= P_{H_2} \cdot \left(0.5P_{H_2}^{-1} - \frac{0.5K_{H_2}^{0.5}P_{H_2}^{-0.5}}{K_{H_2}^{0.5}P_{H_2}^{0.5}+1}\right) = 0.5 - \frac{0.5K_{H_2}^{0.5}P_{H_2}^{0.5}}{K_{H_2}^{0.5}P_{H_2}^{0.5}+1} \qquad \text{(Eqn. S16)}$$

By substituting Eqn. S14 into Eqn. S16, the reaction order in H_2:

$$n_{H_2} = 0.5 - 0.5\theta_H \qquad \text{(Eqn. S17)}$$

This model has the constraint of $n_{H_2} \leq 0.5$, in contrary to our experimental measurement (n_{H_2} = 1). Therefore, the 2nd H addition instead of the first one is the rds.

Reaction order in furfural = $\frac{\partial \ln r}{\partial \ln C_F}$

$$= \frac{\partial C_F}{\partial \ln C_F} \cdot \frac{\partial \ln r}{\partial C_F} = C_F \cdot \frac{\partial \ln r}{\partial C_F} = C_F \cdot \frac{\partial}{\partial C_F} \ln\left[\frac{k_1 K_{H_2}^{0.5}P_{H_2}^{0.5}K_F C_F}{(K_{H_2}^{0.5}P_{H_2}^{0.5}+1)(K_F C_F+1)}\right]$$

$$= C_F \cdot \frac{\partial}{\partial C_F}\left[\ln\left(k_1 K_{H_2}^{0.5}P_{H_2}^{0.5}K_F C_F\right) - \ln\left(K_{H_2}^{0.5}P_{H_2}^{0.5}+1\right) - \ln(K_F C_F+1)\right]$$

$$= C_F \cdot \left(\frac{1}{C_F} - \frac{K_F}{K_F C_F+1}\right) = 1 - \frac{K_F C_F}{K_F C_F+1} \qquad \text{(Eqn. S18)}$$

By substituting Eqn. S14 into Eqn. S18, the reaction order in furfural:

$$n_F = 1 - \theta_F \qquad \text{(Eqn. S19)}$$

Supplementary References

1 Yang, G. J. *et al.* The nature of hydrogen adsorption on platinum in the aqueous phase. *Angew. Chem. Int. Edit.* **58**, 3527-3532, doi:10.1002/anie.201813958 (2019).

2 Sander, R. Compilation of Henry's law constants (version 4.0) for water as solvent. *Atmos. Chem. Phys.* **15**, 4399-4981 (2015).

3 Kühne, R., Ebert, R.-U. & Schüürmann, G. Prediction of the temperature dependency of Henry's law constant from chemical structure. *Environ. Sci. Technol.* **39**, 6705-6711 (2005).

4 Singh, N. & Campbell, C. T. A simple bond-additivity model explains large decreases in heats of adsorption in solvents versus gas phase: a case study with phenol on Pt (111) in water. *ACS Catal.* **9**, 8116-8127 (2019).

5 Osborne, N. S. Measurements of Heat Capacity and Heat of Vaporization of Water in the Range 0℃ to 100℃. *J. Res. Nat. Bur. Stand.* **23**, 197-260 (1939).

6 Allen, K., Lewis, D. & Pankhurst, K. Adsorption of water vapour on palladium. *J. Chem. Soc. A: Inorg. Phys. Theor.* 3028-3031 (1971).

7 Israelachvili, J. N. *Intermolecular and surface forces*. (Academic press, 2015).

8 Davey, W. P. Precision measurements of the lattice constants of twelve common metals. *Phys. Rev.* **25**, 753 (1925).
